# *Cis*-Acting Polymorphisms Affect Complex Traits through Modifications of MicroRNA Regulation Pathways

**DOI:** 10.1371/journal.pone.0036694

**Published:** 2012-05-11

**Authors:** Matthias Arnold, Daniel C. Ellwanger, Mara L. Hartsperger, Arne Pfeufer, Volker Stümpflen

**Affiliations:** 1 Institute of Bioinformatics and Systems Biology, Helmholtz Zentrum München, German Research Center for Environmental Health, Neuherberg, Germany; 2 Chair of Genome-Oriented Bioinformatics, Technische Universität München, Center of Life and Food Science, Freising-Weihenstephan, Germany; 3 Institute for Human Genetics, Technische Universität München, Munich, Germany; 4 Institute of Human Genetics, Helmholtz Zentrum München, German National Research Center for Environmental Health, Neuherberg, Germany; 5 Institute of Genetic Medicine, European Academy Bozen/Bolzano (EURAC), Bolzano, Italy - Affiliated Institute of the University Lübeck, Germany; University of Illinois at Chicago, United States of America

## Abstract

Genome-wide association studies (GWAS) have become an effective tool to map genes and regions contributing to multifactorial human diseases and traits. A comparably small number of variants identified by GWAS are known to have a direct effect on protein structure whereas the majority of variants is thought to exert their moderate influences on the phenotype through regulatory changes in mRNA expression. MicroRNAs (miRNAs) have been identified as powerful posttranscriptional regulators of mRNAs. Binding to their target sites, which are mostly located within the 3′-untranslated region (3′-UTR) of mRNA transcripts, they modulate mRNA expression and stability. Until today almost all human mRNA transcripts are known to harbor at least one miRNA target site with an average of over 20 miRNA target sites per transcript. Among 5,101 GWAS-identified sentinel single nucleotide polymorphisms (SNPs) that correspond to 18,884 SNPs in linkage disequilibrium (LD) with the sentinels (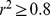) we identified a significant overrepresentation of SNPs that affect the 3′-UTR of genes (OR = 2.33, 95% CI = 2.12–2.57, 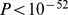). This effect was even stronger considering all SNPs in one LD bin a single signal (OR = 4.27, 95% CI = 3.84–4.74, 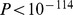). Based on crosslinking immunoprecipitation data we identified four mechanisms affecting miRNA regulation by 3′-UTR mutations: (i) deletion or (ii) creation of miRNA recognition elements within validated RNA-induced silencing complex binding sites, (iii) alteration of 3′-UTR splicing leading to a loss of binding sites, and (iv) change of binding affinity due to modifications of 3′-UTR folding. We annotated 53 SNPs of a total of 288 trait-associated 3′-UTR SNPs as mediating at least one of these mechanisms. Using a qualitative systems biology approach, we demonstrate how our findings can be used to support biological interpretation of GWAS results as well as to provide new experimentally testable hypotheses.

## Introduction

The state of knowledge regarding development, progression and inheritability of human diseases has long since outrun the classical understanding. There are only few disorders where monogenic causes could be determined. Also, the genetic links to disease incident are missing to a substantial part and the complexity of pathways encompassing pathogenic effects can still not be limited upwards. In recent years, more and more evidence is emerging that microRNAs (miRNAs), a class of small non-coding RNAs, play an important role in human traits. Databases collecting information on miRNAs mediating human disease such as miR2Disease [1] or PhenomiR [2] list several hundred miRNAs with proven roles in way above 100 human diseases.

MiRNAs are key posttranscriptional regulators of most known cellular processes and have been associated with cell fate decision, development, and stress response. Additionally, miRNAs have been identified to be usable as biomarkers for human diseases [2]–[5]. With growing knowledge on their targets, which are believed to make up more than 60% of all protein-coding genes [7], new regulatory and disease-mediating gene networks were discovered [8]–[11]. Because of the ability of single miRNAs to regulate not only one but up to several hundred genes, they depict a promising drug target for disease pathways involving multiple genes. With the recent advances of the crosslinking immunoprecipitation (CLIP) technology, it has become feasible to experimentally determine miRNA-target interactions and the exact binding sites of the RNA-binding proteins (RBPs) on transcriptome scale [12]–[15].

As soon as the importance of miRNA functioning for human health was realized, approaches were undertaken with the objective to identify potential interrelations of miRNA dysregulation and genetic variation. However, until now neither the data on trait-associated polymorphisms nor experimentally verified miRNA targeting information provided a sufficient basis for such analyses. For instance, mutations in the 3′-UTR, the major target of miRNA-mediated regulation, have long been (and are still) neglected for the most part in association studies. Therefore, only few particular cases of polymorphisms affecting miRNA regulation pathways have been identified, yet [16], [17]. Up to now such studies are often limited to effects on (mostly predicted) miRNA target sites [17], [18]. However, the 3′-UTR harbors several other functional elements which may, if affected, also mediate disruption of miRNA regulation pathways. It has been assumed, for instance, that the loss of a polyadenylation (poly(A)) signal can cause genetic diseases by non-specific degradation of the mRNA [19]. Recent experiments suggest that this effect may be based on a functional correlation of poly(A) signal efficiency and miRNA-mediated repression [20]. Further, the structural accessibility of an RNA region is an important feature for the binding affinity of RNA-induced silencing complex (RISC) target sites [21]. It has been shown that mutations in RNAs have large local as well as global structural effects [22] and that altered target accessibility can reduce miRNA-mediated posttranscriptional repression to a scale comparable to that of mutations disrupting miRNA recognition element (MRE) sequence complementarity [23]. Finally, polymorphisms affecting splice sites can lead to radical sequence changes increasing susceptibility to diseases, an effect which is suspected to be partly due to altered translation efficiency of the affected mRNA [24] - which is characteristic for miRNA functioning.

The success of genome-wide association studies (GWAS) in determining the genetic causes of some common diseases led to an immense increase of the efforts put on screening common alleles for disease involvement. Since then, more and more loci associated with trait susceptibility are detected. GWAS can, however, neither identify causal genes in associated loci nor provide functional mechanisms behind observed association signals. Consequently, many identified GWAS signals are awaiting mechanistic characterization and the rate at which GWAS signals are currently discovered necessitates systematic and scalable functional approaches [25].

When it became clear that only few diseases are due to common risk alleles, sequencing endeavors have been performed to identify putatively rare variants hiding behind the signals detected in GWAS. In some cases these approaches successfully identified rare causative variants predisposing to disease. However, the expected breakthrough failed to appear as in several cases sequencing of risk loci did not provide further knowledge [26]–[28]. With the situation additionally compounded by the fact that most GWAS-identified alleles are located within non-coding regions, now one focus is laid on the identification of regulatory variants. In this work, we concentrate on the influence of GWAS-identified variants on miRNA-mediated *cis*-acting regulatory effects.

We assess the potential impact of trait-associated SNPs on miRNA regulation pathways using publicly available GWAS data [29], [30]. We describe potential posttranscriptional effects of SNPs by systematically investigating mutations within the 3′-UTR of human transcripts for interference with poly(A) signals, 3′-UTR splicing, 3′-UTR secondary structure changes and MREs. Using the example of *rs10923* in the 3′-UTR of *SMC4* we show how our findings can be utilized in the biological interpretation of GWAS results in a systems biological manner.

## Results

### Trait-associated Variants are Significantly Overrepresented in the 3′-UTR

We compared the amount of trait-associated variants within the predefined five function classes of SNPs (intergenic, intronic, 5′-untranslated region (5′-UTR), coding sequence (CDS), and 3′-UTR) to examine a potential location bias of these markers. Of 18,884 SNPs contained in the extended GWAS-SNP set (GWAS-SNPs and their highly correlating LD partners, 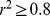), we found 436 to be located in the 3′-UTR of 326 human genes (OR = 2.331, 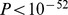, referred to as 3′-UTR SNPs). This is a higher enrichment than for sentinel SNPs only (OR = 2.059, 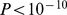). We calculated the probability to get an 3′-UTR enrichment this strong in a random subset of HapMap-SNPs [31]–[33] of comparable size which confirmed significance (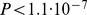). For further validation of this enrichment we looked at the dependencies between the OR and the minor allele frequency (MAF) as well as different *r* ^2^ thresholds.

**Figure 1:**
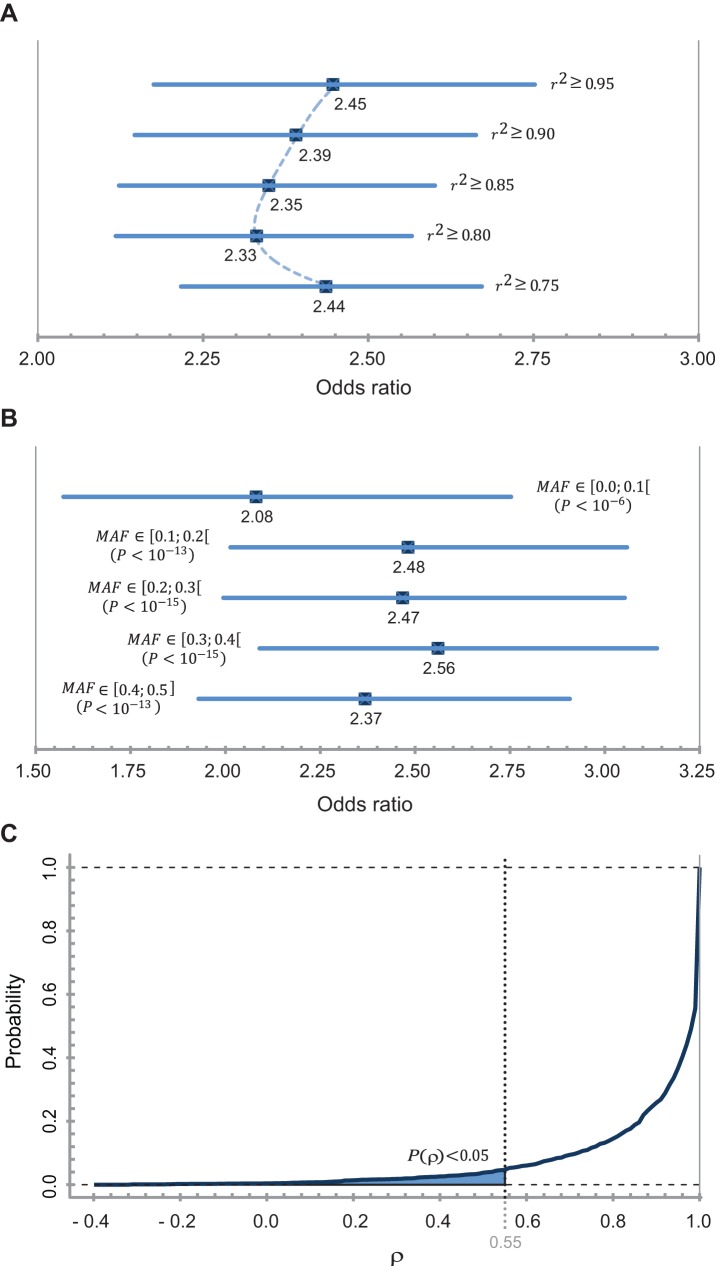
Statistical analysis of 3′-UTR enrichment values and determination of the folding correlation coefficient threshold. **A:** SNP enrichment in the 3′-UTR in dependency of different LD thresholds. Displayed are the ORs and confidence intervals for five cut-offs. Accumulative 3′-UTR SNP sets were calculated. The fitted distribution (dashed line) points out the stabilization of the OR around a threshold of 0.8. **B:** SNP enrichment in the 3′-UTR in dependency of the minor allele frequency. Displayed are the ORs and confidence intervals for the 5 different MAF bins. SNP counts were compared within the respective bins. **C:** Probability distribution of correlation coefficients (*p*) between wild-type and mutated structures of RBP-binding regions. Below a cut-off for the correlation coefficient of 0.55 (displayed in gray) the probability to observe a change of RNA secondary structure of this scale by chance amounts to less than 5%.

When we adjusted for *r* ^2^ in the extension of the GWAS-SNPs, we found that the distribution of ORs locally stabilizes around a threshold of 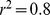 **(** **Figure 1A** **)**. This limit thus seems to fit the data better than more rigid thresholds and was therefore chosen in this work. To rule out a false increase of the enrichment due to correlating SNPs in the same 3′-UTRs (SNP-gene ratio 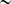
1.34) we binned the complete HapMap-SNPs into blocks with an all-vs.-all 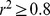. More than one million HapMap SNPs were binned together in about 371,000 blocks containing more than two SNPs. The remaining SNPs only showed pairwise or no LD at the chosen threshold. When we included all SNPs after binning, the OR for 3′-UTR enrichment was even greater than without binning (OR = 4.27, 95% CI = 3.84–4.74, 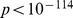). Considering only the SNPs within the 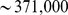 blocks the enrichment still holds significance (OR = 1.828, 95% CI = 1.63–2.04, 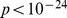). As about 10% of the 3′-UTR SNPs are not contained in theses blocks, we suggest that this value presents an underestimation of the actual enrichment. The reason for the stronger enrichment after binning is that the SNP count within the LD blocks depends on the location of the SNPs. While intronic and intergenic SNPs are reduced to less than 35% (block-SNP ratio) by binning, SNPs in exonic regions present less extensive LD patterns (reduction only to about 81%).

By analyzing the MAF, we found the extended GWAS-SNPs to hold a commonly higher MAF than the HapMap-SNPs, regardless of their chromosomal location. However, the comparison of the MAF distribution of the 3′-UTR SNPs to the MAF distribution of other extended GWAS-SNPs revealed a slight trend of 3′-UTR SNPs towards moderate MAF frequencies between 0.1 and 0.4. This trend becomes more explicit when comparing the 3′-UTR SNPs to the combined extended GWAS-SNPs in the other two exonic regions (i.e. 5′-UTR and CDS). In comparison, 3′-UTR SNPs show underrepresentation of the intervals 0.0–0.1 (OR = 0.88), 0.2–0.3 (OR = 0.70) and 0.4–0.5 (OR = 0.78) whereas the other two intervals are significantly (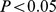) overrepresented (OR_0.1–0.2_ = 1.40 and OR_0.1–0.2_ = 1.59). To investigate if 3′-UTR SNP enrichment values hold only for specific MAFs, we recalculated the ORs against the HapMap-SNP set in dependency of the MAF. The ORs resulting for the five MAF intervals follow roughly the pattern of over−/underrepresentation observed in the comparison with the other extended GWAS-SNPs but never lose significance or fall below an OR of 2.0 **(** **Figure 1B** **)**. 

### Enrichment Analysis Indicates Gene Involvement in Lipid Metabolism

To investigate whether the 326 genes affected by 3′-UTR mutations share common characteristics, we performed gene enrichment analyses with respect to disease involvement and functional annotations.

By mapping the traits associated with 3′-UTR variants to MeSH terms we retrieved a total of 49 observed disease classes. The most abundant categories were immune system diseases, mental disorders, digestive system diseases, nervous system diseases, and neoplasms. The distribution of the 3′-UTR SNPs within these disease classes showed no significant overrepresentation compared to the count of studies performed for the single disorders in the GWAS Catalog. When comparing the number of 3′-UTR SNPs per disease to the count of all non-3′-UTR extended GWAS-SNPs, we found only lipid concentrations to be significantly (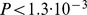) enriched in the 3′-UTR set.

Gene set enrichment analysis (GSEA) revealed only four significantly enriched (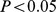 after Bonferroni correction) functional annotations in this set: lipid metabolism, axon growth, activation of the immune response/inflammation (9 terms), and regulation of/response to cell signaling (10 terms). Using DAVID [34], [35], we also checked for overrepresentation of disease terms not limited to the GWAS Catalog and found three enriched terms (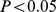 after correction): Dyslipidemia (background set: Online Mendelian Inheritance in Man database), neurological diseases, and infections (background set: Genetic Association Database [36]).

### Evidence for Impact on miRNA-mediated Regulation

**Figure 2:**
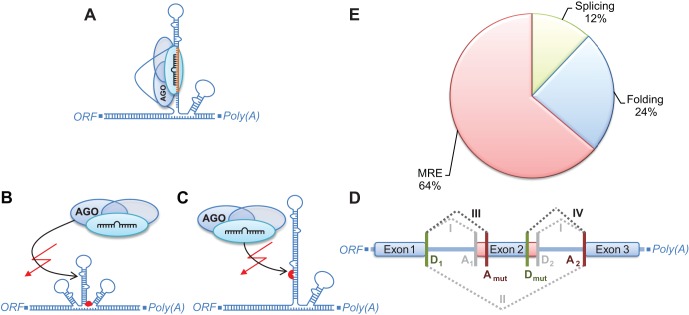
Mechanisms mediated by 3′-UTR SNPs affecting miRNA targeting. **A:** Regular binding of the RISC to the target mRNA. **B:** Binding of the RISC and, thus, miRNA-mediated silencing is inhibited by a change in RNA secondary structure. **C:** A mutation within the MRE seed site disrupts the ability of a certain miRNA to target a transcript. Here, the opposite effect also applies, i.e. a new MRE seed site is formed by a polymorphism which enables targeting by a miRNA usually not controlling the respective transcript. **D:** Altered splicing by acceptor or donor splice site gain. The existing splice variants (I and II, grayed) are extended by mutationally introduced additional splice variants: (III) A present acceptor site (A_1_) is substituted by a new acceptor site (A*_mut_*), and (IV) a naturally occurring donor site (D_2_) is replaced by a new donor site (D*_mut_*). Both effects may lead to a considerable loss of exon sequence (displayed in red) and, thus, RISC binding sites. **E:** The percentages of classified SNPs mediating the single mechanisms. The greatest amount of functionally annotated 3′-UTR SNPs directly affect MRE sequences, followed by SNPs changing the RNA secondary structure and SNPs with an predicted effect on 3′-UTR splicing.

The efficacy of a miRNA to control target mRNA translation relies, among others, on three sequence-based features: correct mRNA processing, presence of a functional MRE, and accessibility of the RISC binding site. To find out to which extent trait-associated SNPs in 3′-UTRs affect miRNA functioning, we examined four mechanisms potentially compromising these features **(** **Figure 2** **,** **Table 1**. This analysis was limited to transcripts featuring both 3′-UTR SNPs and validated RISC binding sites. The according data set contained 288 SNPs on 409 transcripts and 219 genes, respectively. Firstly, we investigated potential effects of SNPs on mRNA processing by interfering with poly(A) signals. We found four SNPs affecting hexamers with a sequence characteristic for poly(A) signals, however, none of these hexamers was located near a validated poly(A) site. A functional effect of those variants on mRNA processing thus seems unlikely. Secondly, we analyzed the impact on mRNA splicing. We identified seven SNPs (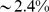) predicted to interfere with RNA splice sites **(** **Figure 2D** **, Table S1)**. Six of those are predicted to create new acceptor sites and one to create a new donor site **(** **Figure 2D** **)**. SNPs interfering with splice sites located at an exon/intron or intron/exon border as annotated in RefSeq were not found. The probability to observe such an effect by chance is 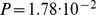 for acceptor sites and 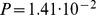 for donor sites. In all seven cases, the predicted gain of splice sites results in exon shortening, leading to a noticeable loss (46% on average) of RISC binding sites in the accordant transcripts. Thirdly, we searched for SNPs which may affect the secondary structure of the 3′-UTR proximal to a validated RISC binding site causing an altered accessibility of the region. This resulted in 14 SNPs (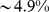) predicted to affect the binding affinity of the RISC through changed secondary structure of the 3′-UTR **(** **Figure 2B** **, Table S2)**. Fourthly, we examined direct effects of SNPs on MREs located in validated RISC binding sites. We found 22 SNPs (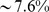) disrupting MREs **(** **Figure 2C** **, Table S3)**, and 28 SNPs (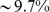) creating new MREs **(** **Figure 2C** **, Table S4)**. The overlap between the SNP sets creating and disrupting MREs, i.e. SNPs substituting the MRE of one miRNA by a MRE of another miRNA, amounts to 13 variants. Accordingly, a total of 37 unique SNPs (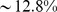) directly affect MREs. The probability of obtaining these amounts of SNPs affecting MREs randomly was 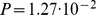 (disruption) and 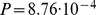 (creation). Additionally, we found that only 11% of SNPs enhancing (i.e. extending an already existing seed match) or creating a MRE were conserved across mammals which was a lower fraction than for SNPs causing one of the other effects (folding = 29%, splicing = 29%, MRE disruption = 27%).

**Table 1:** SNPs affecting functional elements with *cis*-regulatory effects on miRNA regulation.

SNP	Gene	Effects	Traits
rs1121	PDXDC1	MRE creation	Height
rs4564	DLD	MRE disruption	Ulcerative Colitis
rs6706	TRIP6	MRE disruption	Resting Heart Rate
rs7089	TMUB2	MRE disruption; MRE creation	Bone Density
rs7097	POLR1D	MRE creation	Large B-Cell Lymphoma
rs7118	ZFP90	MRE disruption; MRE creation	Ulcerative Colitis
rs7119	HMG20A	MRE disruption	Type 2 Diabetes
rs7371	GNAI3	Acceptor gain	Major Depressive Disorder
rs7444	UBE2L3	Folding	Crohn’s Disease; Systemic Lupus Erythematosus
rs8523	ELOVL2	MRE disruption; MRE creation	Phospholipid levels
rs9253	MEAF6	MRE disruption	Hematological Phenotypes
rs9927	PYGB	MRE creation	Liver Enzyme Levels
rs10923	SMC4	MRE disruption; MRE creation	PBC
rs11700	E2F4	MRE creation	Coronary Heart Disease
rs12439	CLIC4	MRE disruption; MRE creation	Height
rs12916	HMGCR	MRE creation	Cholesterol levels; Metabolic Traits
rs12956	RYBP	Folding	Height
rs13099	TMED10	Folding	Height
rs42038	CDK6	Folding; Acceptor gain	Height
rs42039	CDK6	MRE creation	Rheumatoid Arthritis
rs232775	MYSM1	MRE creation	Diabetic Retinopathy
rs699779	NOTCH2	Acceptor gain; MRE disruption	Type 2 Diabetes
rs823136	RAB7L1	MRE creation	Parkinson’s Disease
rs835575	NOTCH2	Folding; MRE disruption; MRE creation	Type 2 Diabetes
rs835576	NOTCH2	MRE disruption; MRE creation	Type 2 Diabetes
rs1045100	ATG16L1	MRE disruption; MRE creation	Crohn’s Disease
rs1045407	ZNF678	Folding; MRE creation	Height
rs1046917	FN3KRP	Folding	Glycated Hemoglobin Levels
rs1047440	CEP120	MRE disruption; MRE creation	Body Mass Index
rs1058588	VAMP8	MRE disruption	Prostate Cancer
rs1379659	SLIT2	MRE disruption	Echocardiographic Traits
rs2032933	RMI2	MRE creation	Celiac Disease
rs2071518	NOV	MRE creation	Blood Pressure
rs2077579	DDX6	Folding	PBC
rs2229302	HOXB2	MRE disruption; MRE creation	Primary Tooth Development
rs2244967	VSTM4	Acceptor gain	Serum Uric Acid
rs2282301	RIT1	Folding	Conduct Disorder
rs2293578	SLC39A13	MRE creation	Body Mass Index
rs2564921	RFT1	Folding	Height
rs3816661	CD276	MRE disruption	Liver Enzyme Levels
rs3821301	TANC1	Folding	Sudden Cardiac Arrest
rs4770433	SACS	MRE disruption	Protein Quantitative Trait Loci
rs4819388	ICOSLG	Folding; MRE creation	Celiac Disease
rs4973768	SLC4A7	Donor gain	Breast Cancer
rs6722332	WDR12	Acceptor gain	Coronary Heart Disease; Myocardial Infarction
rs7350928	KIAA1267	MRE disruption; MRE creation	Parkinson’s Disease
rs7528419	CELSR2	Acceptor gain	Cholesterol levels; Metabolic traits; Cardiovascular Disease; Myocardial Infarction; Response to Statins
rs8176751	ABO	MRE creation	Hematological Phenotypes
rs10892082	PAFAH1B2	Folding	Protein QTLs; Triglyceride Levels
rs11067231	MMAB	MRE creation	Cholesterol levels
rs11542478	FAM110C	Folding	Information Processing Speed
rs11713355	SLC6A6	MRE disruption; MRE creation	Cognitive Performance
rs17574361	KIAA1267	MRE disruption; MRE creation	Parkinson’s Disease

The first column gives the rs-number of the SNPs, in the second column the HGNC symbol of the affected genes are listed and the third column describes the functional mechanisms which could be assigned to the SNPs. The last column contains all traits associated with the respective SNP.

### *SMC4 –* from Primary Biliary Cirrhosis to Cancer

The autoimmune disease primary biliary cirrhosis (PBC) is associated with the damaging of the small bile ducts and is mediated by auto-antibodies [37]–[39]. The autoimmune response caused by those antibodies leads to inflammation followed by aggregation of dead cells. Apoptosis is induced, among other things, by reactive molecules effecting DNA damage [40], [41] leading to a build-up of scar tissue (i.e. cirrhosis). The genetic background of the disease was focus of a recent GWAS [42], however, the rationale of the study was restricted to the major histocompatibility complex and interleukins. Causative variants could not be identified so far. One of the SNPs (*rs4679904*) reported in the study is in high LD (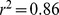) with the 3′-UTR SNP *rs10923* in *SMC4* which is part of the *Condensin I* complex [43]. Interestingly, DNA repair genes such as *PARP1* and *XRCC1* are over-expressed in cirrhotic tissue and are in this context hypothesized to feature pathogenic effects [44]. For full functioning of the *PARP1-XRCC1* complex in single-strand break repair, an association with the *Condensin I* complex is established [45].

**Figure 3:**
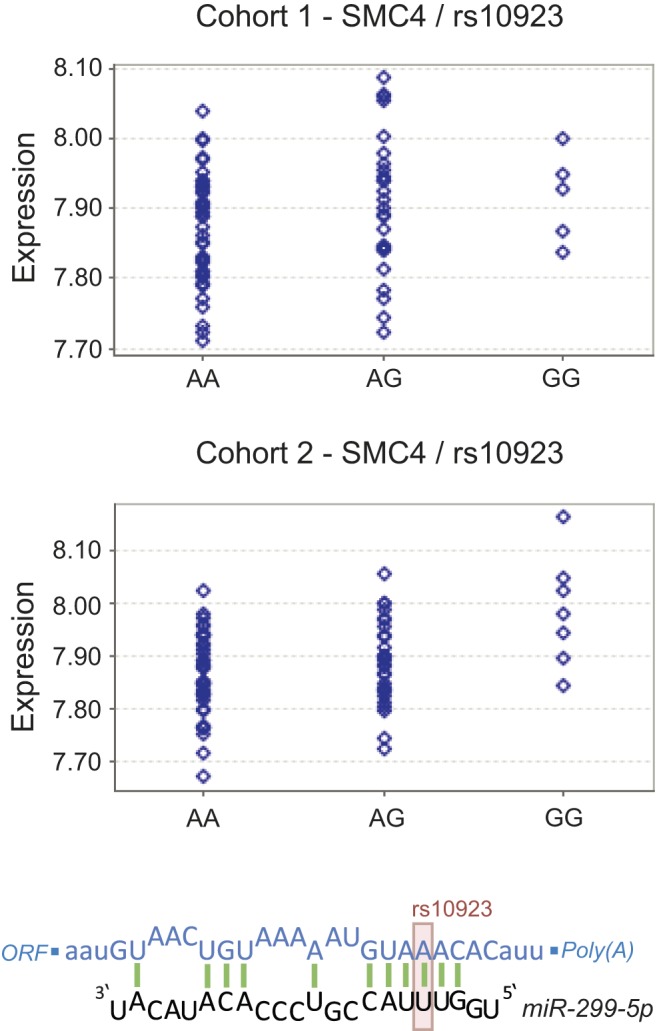
Impact of the SNP *rs10923* on miRNA-mediated repression of *SMC4*. Shown is the mRNA:miRNA duplex for the reference allele of *rs10923* (lower part). The minor allele of the SNP (position adumbrated by the light red box) disrupts the seed complementary region. In the upper part of the figure, the expression pattern of *SMC4* in lymphoblastoid cells is illustrated. The minor G allele of the polymorphism is significantly (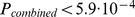) linked to an increased abundance of *SMC4* transcript. For the illustration of expression values Genevar output was adapted [89].

**Figure 4:**
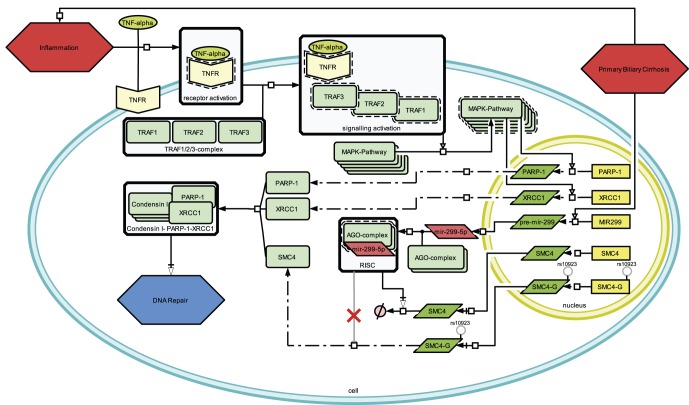
Impact model of mutated SMC4 in primary biliary cirrhosis. Inflammation follows the autoimmune response leading to the activation of the *MAPK*-pathway via signal molecules as e.g. *TNF-alpha*. Transcription factors activated as downstream effect of *MAPK* activation lead to over-expression of DNA repair genes. The in PBC over-expressed *hsa-mir-299-5p* is hypothesized to target *SMC4* at the seed complementary region where *rs10923* is located. With the major allele, *SMC4* is silenced, whereas the mutated *SMC4*-G cannot be bound by *hsa-mir-299-5p* and therefore is translated without interference. This results in the more frequent association of the *Condensin I-PARP1-XRCC1* complex contributing to disturbed DNA repair in cirrhosis tissue.

The SNP *rs10923* is localized within an experimentally validated RISC binding site [14] and lies directly in the seed complementary region of *hsa-mir-299-5p*, a miRNA that has been shown to be up-regulated in PBC patients [46]. The minor G allele of *rs10923* disrupts the 6*mer
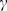
* seed complementary region **(** **Table 1** **)** and thus the ability of the miRNA to bind the transcript **(** **Figure 3** **)** [47]. Although we also observe the gain of a new MRE when introducing the minor allele of the SNP **(** **Table 1** **)**, expression data from the MuTHER study [48] supports the hypothesis of deactivated miRNA-control as it shows significant (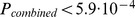) association of the G allele with increased *SMC4* expression in lymphoblastoid cell lines. We suggest that, by this process, *rs10923* contributes to the phenomenon of DNA repair perturbation in cirrhosis **(** **Figure 4** **)** [44]. Beyond that this may indicate a rate-limiting character of *SMC4* in the generation of the *Condensin I-PARP1-XRCC1* complex. Furthermore, PBC patients present elevated risk to develop different types of cancer [49], [50] representing a potential explanation for the deranged DNA repair functionality in the disease. In cancer development and progression, up-regulated DNA damage response is associated with mutagenesis and resistance to radio- and chemotherapy [41], [51]–[53].

## Discussion

Although thousands of SNP-trait associations have been published and the applicability of GWAS is broadly appreciated, the associations identified by GWAS hold predictive power for only a small fraction of disease incidence so far [25], [54]. Finding the causative variants predisposing individual disease risk therefore presents the main dilemma of GWAS. As long as comprehensive sequencing data is missing for most associated loci, frameworks are needed providing a first characterization of potential disease-mediating mechanisms.

Here, we suggest that associated common or unknown rare variants in the 3′-UTR depict posttranscriptionally regulatory variants which, to differing extent, affect disease development and progression by interfering with miRNA regulatory networks. The differing impact of 3′-UTR SNPs can be explained with two facts: first, human mRNAs are mostly targeted by multiple miRNAs at different target sites which may allow for compensating the loss of a single binding site; and second, the extent to which mRNAs are silenced by miRNAs depends on multiple factors such as tissue-specific miRNA expression, the binding affinity of the RISC and the degradation rate of the mRNA [55], [56]. Thus, unlike mutationally driven alterations of the amino acid sequence of a protein or disruption of a transcription factor binding site in promoter or enhancer regions, interferences with miRNA regulation may show tissue-specific effects sizing from low or not distinguishable to high or even causative.

Based on recently available data, our results clearly show that several lines of evidence become obvious that miRNAs play an important role in genetically determined posttranscriptional disease development and progression. There is no evidence for considerable direct interference of miRNA processing: only one SNP (*rs2168518*) was found to be located in the hairpin of *hsa-mir-4513*. But we observed a significant overrepresentation of trait-associated SNPs in the 3′-UTR that strongly suggests a functional coherence between genetic variants and miRNA regulation pathways on the *cis*-regulatory level.

Several studies assessing the disease-mediating power of miRNAs confirmed their involvement in the mediation of diverse traits [3], [57], [58] suggesting that traits associated with 3′-UTR SNPs belong to heterogeneous disease classes. We observed a similar trend as our results showed a correlation between the number of traits belonging to the distinct disease classes and the number of published studies on the corresponding traits in the GWAS catalog [30]. This indicates that with an increasing number of studies on other traits the count of disease classes associated with 3′-UTR SNPs will increase, too. Moreover, an uniform number of studies on the traits should lead to an equal distribution of the represented disease classes. Of a total of 49 disease classes assigned to the 3′-UTR SNPs, only lipid concentrations showed a significant enrichment within the phenotypes investigated by GWAS.

The functional enrichment analysis revealed that genes having a 3′-UTR SNP are involved in a wide range of cellular processes. The most intriguing finding is the replication of the enrichment of lipid traits by GSEA which, additionally, detected an enrichment of the lipid metabolism on the level of gene function. The enrichment of terms regarding the regulation of the immune response and of inflammatory processes, on the contrary, reflects the phenotype bias of GWAS towards autoimmune and inflammatory diseases [59]. The other overrepresented terms are connected to multiple downstream effects indicating a major participation of the genes in the initiation and regulation of cellular pathways.

Despite the fact that we could not find an interrelation of polymorphisms and poly(A) signals, our extensive analysis of the potential impact of GWAS-identified SNPs on functional elements in the 3′-UTR revealed several mechanisms whereby variants may affect miRNA-mediated regulation. The smallest fraction of potentially functional 3′-UTR SNPs affects 3′-UTR splicing. These SNPs are predicted to mediate miRNA target site loss, mostly through the gain of acceptor splice sites (donor splice site gain only occurred once) resulting in shortened 3′-UTRs. That altered splicing is the rarest found mechanism can be explained by the huge impact it mediates on miRNA targeting which manifests in the high fraction of target site loss (46% on average) for the affected transcripts. The second mechanism is the SNP-mediated alteration of RNA secondary structure of a RISC binding region. The impact of RNA folding on the binding affinity of RBPs has already been described [21], [23]. However, the extent to which this phenomenon translates into miRNAs mediating human disease development is unknown. With our results we provide a first data basis on potentially pathogenic RNA structural changes which may serve as a starting point to investigate this matter further. The third and most abundant mechanism we could identify is the direct alteration of MRE sequences. We find not only that GWAS-identified markers in the 3′-UTR show a significant enrichment within MREs, but also identify a novel scenario of how miRNA dysregulation may take effect: the substitution of the recognition element of one miRNA by one of another miRNA. While a disruption (or creation, respectively) of a MRE enables a rather straightforward rationale, that is the tissue-specific repression (or enhancement, respectively) of miRNA regulation, this scenario makes interpretation rather complex. Such a substitution may imply concurrent but simultaneously diverging effects in different tissues, depending on the respective expression patterns of the two miRNAs, possibly leading to systemic disturbances of several cell types. The overlap between the two sets of SNPs which disrupt and create MREs amounts to 13 polymorphisms and constitutes more than one third of the set of variants affecting MREs - which is a surprisingly high number. We believe that the transcripts affected by a SNP mediating this effect may present quite interesting targets for further studies. Moreover, this highest fraction (39%) of effective 3′-UTR SNPs shows a low conservation indicating that the creation of a MRE may be an abundant process of functional SNPs.

In general, our findings indicate that miRNAs play an important role in genetic variants causing trait development. Therefore, novel aspects not only for the interrelations in pathogenic disturbance of cellular processes, but also for the coherence of different traits can be addressed. For instance, differential tissue-specific expression patterns of miRNAs in combination with genetic variants may shed light on the still unknown functions driving the same cellular pathways to feature different effects in diseased and healthy individuals. Thus, closing the gap between trait-associated mutations and impaired miRNA-mediated gene regulation may lead the way to a new understanding of the interconnection of those functional entities.

We found 326 candidate genes, and on the example of *SMC4* we show that the incorporation of miRNA related information can be used to construct models of potential disease-driving mechanisms in a systems biological fashion. In this case, we present a very simple one-gene/one-miRNA scenario. However, up to now *SMC4* and PBC have never been connected, as well as an association of PBC and cancer only exists in clinical ratios. But our suggestion of a highly active, *SMC4*-dependent DNA repair machinery is not only a likely cellular response mechanism to apoptosis signals in cirrhotic tissue. It also provides the link to cell transformation and cancerogenesis. Therefore, in the context of miRNA biology plausible coherences can be established for common variants with unknown effect. This also points out that, beyond statistical evaluations, case-specific detailed analyses may greatly outrange global approaches in the biological interpretation of GWAS results.

Our findings highlight numerous starting points for biomedical research. To advance the potential of conclusion drawing in GWAS results interpretation, it may be invaluable to overcome the GWAS bias towards the coding sequence and extend approaches based on 3′-UTR mutations. Conversely, more comprehensive data on miRNA binding sites might enhance our understanding of miRNA regulation functioning. It may also be of interest to investigate the most suspicious cases described in this work further. Moreover, determination of miRNA regulation via RISC binding to other mRNA locations than the 3′-UTR might shed light on the effect of coding-synonymous trait-associated alleles. The combination, i.e. extensive measurements of miRNA-mediated gene regulation in patients with traits for which a plausible model of miRNA involvement can be created in the context of associated mutations in the 3′-UTR, may further provide new perspectives of disease progression.

 But the implications of our findings go beyond GWAS and miRNAs. Considering the advances made in the exploration of the still poorly understood elements of the genome, the impact of the presented results are pointed out. Just recently, the approaches of the modENCODE project [60], [61] led to the validation of the hypothesized hierarchical structure of physical regulatory networks in eukaryotes which are based on a sophisticated interplay of miRNAs and transcription factors. Thus, the important role of this class of non-coding RNAs in the regulatory machinery of the cell is brought out on a large-scale level. If those findings can be transferred from the studied model organisms to human, the analysis of impairment of the transcription factor-miRNA network balance by mutationally altered target site functioning may lead to a completely new definition of genetically predisposed diseases on a RNA-mediated, regulatory basis. Also, miRNAs are only one class of non-coding RNAs which have been proven to feature regulatory power. Interference by e.g. piwi-associated RNAs, small interfering RNAs or large intergenic RNAs which all are incorporated in protein-containing complexes targeting specific genes (especially, their 3′-UTR) will have to be assessed in this context to gather further insights.

## Materials and Methods

### Acquisition of the SNP Data Sets

We downloaded the Catalog of Published Genome-Wide Association Studies [29], which includes information about 5,101 unique SNP-trait associations with a p-value of 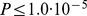 [30]. We refer to this set as GWAS-SNPs. The GWAS-SNPs were extended by highly correlating SNPs with strong LD (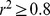) in the HapMap3 CEU panel (Utah residents with northern and western European ancestry from the CEPH collection) [31]–[33]. This set, further referred to as extended GWAS-SNPs, contains 18,884 variants retrieved by using SNAP [62].

As background distribution for localization enrichment we used the 2.7 million SNPs from the CEU panel of the joint HapMap Phases I, II and III (release 27, referred to as HapMap-SNPs) for which genotype information was available [31]–[33]. All SNPs were mapped to official identifiers [63] using SNAP [62] and their genome build NCBI36/hg18 coordinates were retrieved from the UCSC (University of California Santa Cruz) Table Browser [64]. For a background set representative for the HapMap-SNPs with comparable properties as the extended GWAS-SNPs, we randomly selected 5,101 SNPs from the HapMap-SNP set and extended this set, analogous as for the GWAS-SNPs, with SNPs in strong LD using 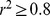. This process was repeated 1000 times. For statistical evaluation of the localization enrichment and the effects on functional elements against this background, we computed the respective statistics for all 1000 sets, fitted a distribution to the resulting values and retrieved the probability to observe the statistic obtained for the extended GWAS-SNPs. As the HapMap-SNPs are a superset of all other SNP sets, i.e. the 1000 random sets and the GWAS-SNPs as well as the extended GWAS-SNPs, we always performed SNP-based annotations for the whole HapMap-SNP set. Thus, we simultaneously retrieved the properties of all other SNP sets.

We mapped all SNPs on genomic locations of protein-coding genes and miRNA genes. The genome annotations were obtained from the UCSC Table Browser [64] based on the NCBI Reference Sequence annotation (genome build NCBI36/hg18) [65] and from miRBase release 18 (genome assembly GRCh37/hg19) [66]–[68]. We used the UCSC liftOver tool [69] to convert the genomic miRNA hairpin coordinates to the NCBI36/hg18 assembly. The chromosomal function of the SNPs was categorized into five classes: intergenic, intronic, 5′-UTR, CDS, and 3′-UTR. To assess differences regarding the MAF in and between the SNP sets we computed the distribution in bins of ten percent range for all SNPs. MAF data was used as given for the HapMap-SNPs. Adjustment for *r* ^2^ values in the localization enrichment analysis was performed accumulatively in 5% steps. For binning of HapMap-SNPs into LD blocks, we searched for all SNP sets with an all-vs.-all 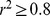. Each SNP was uniquely assigned to a LD block. The localization of the blocks was defined as the subset of the five classes occurring in the annotation of the SNPs contained in the respective bin.

We used the algorithm PhastCons from the PHAST package [70] to calculate the maximum likelihood of a SNP to be conserved across 17 vertebrates. We required a score greater than 0.57 to classify a site as conserved in mammals [71].

### Annotation of RISC-target Regulatory Relationships

To analyze the single nucleotide mutation effect on miRNA targeting, we used the high-throughput transcriptome-wide CLIP-Seq interaction maps describing sites of the RBPs *Argonaute* and *TNRC6* in human *HEK293* cells [14] as provided by the starBase database [72]. The available chromosomal coordinates of the CLIP-Seq clusters were converted to the NCBI36/hg18 genome build and mapped to protein-coding genes according to the NCBI Reference Sequence annotation [65]. The final set contained 139,254 locations of RBP binding regions on 24,442 transcripts. 48% of sites were located within a 3′-UTR.

### Examination of Polyadenylation Signals

We obtained chromosomal positions of poly(A) signals from the PolyA DB for mRNA polyadenylation sites [73], [74]. As the position of poly(A) sites is described to be located 10–30 nucleotides (nt) downstream of the poly(A) signals [73], [75], SNPs within this range site were determined. We then extracted 11 nt long mRNA sequences centered around these 3′-UTR SNPs and examined the sequences for the most abundant poly(A) signal variations according to [75]. We classified a SNP as effecting a poly(A) signal if either creation of a new poly(A) signal sequence or disruption of an existing signal occurs. Classification of a SNP as not effecting a poly(A) signal was carried out if there was no existing signal in the 11 nt sequence for both the wild-type allele and the mutated allele or if the respective allele does not only disrupt a signal but simultaneously creates another poly(A) signal (“synonymous mutation”).

### Determination of Splice Sites

We applied the NNSplice algorithm from the Berkeley Drosophila Genome project [76] to identify changes in splice sites. As input we used a genomic DNA sequence window of 60 nt centered at the SNP position of the wild-type and the mutated type. Predicted splice sites with a likelihood greater than 0.5 were retained neglecting cases with marginal changes [77]. All types of splice site change were considered: loss/gain of splice site and increase/decrease of likelihood. The distance of any splice site change to an exon junction site as defined by the NCBI Reference gene annotation (genome build NCBI36) [65] was computed. We filtered lost acceptor sites or sites exhibiting an increase/decrease in their likelihood if they were located between 100 nt upstream and 10 nt downstream of a reference intron/exon border. Lost donor sites or sites with an increased/decreased likelihood were retained if they were located between 10 nt up- and downstream of a reference exon/intron border. A gain of a completely new splice site was always kept [77].

### Analysis of RNA Structural Properties

To account for structural changes caused by SNPs we used the RNAfold algorithm from the Vienna RNA Package version 1.8.5 [78]. We considered the ensemble of possible RNA conformations by calculation of the partition function and the base pairing probability matrix of the wild-type and the mutated 3′-UTR sequences [79]. The row sums were computed to define a pairing score for each nucleotide. We extracted a 41 nt long score vector centered at an RBP:RNA interaction site for the wild-type and the mutated structure. The linear correlation between both structures was measured by the Pearson product-moment correlation coefficient [80]. Since a transcript can hold several RBP-binding sites we selected for each SNP the smallest correlation coefficient per transcript. To evaluate the significance of a change in the RNA structural ensemble we calculated the minimal correlation coefficient for all SNPs of all 1000 random samples. Based on this distribution we determined a correlation coefficient of 0.55 having a probability of less than 5% for a type I error **(** **Figure 1C** **)**. All SNPs inducing a minimal correlation coefficient of less than 0.55 between wild-type and mutated structure were filtered.

### Identification of Altered MREs

 To find all possible MREs we searched for sites complementary to a canonical miRNA seed sequence [47] within the wild-type and mutated 3′-UTRs. The seeking for short sequence matches may yield a plethora of putative target sites with a high false positive rate. The CLIP-seq methods have been shown to significantly reduce the fraction of false positive MREs [14], [15]. Thus, we classified MREs as functional if they were located within a distance of 21 nt to the center of a RBP interaction site [14]. To additionally reduce the rate of false positives we required at least one miRNA sequence read as reported in the accordant Clip-Seq experiment. Further, the enrichment of MREs of each miRNA within RISC-binding regions was calculated. To identify MREs disrupted by a SNP we filtered miRNAs of which MREs were significantly overrepresented within RISC-binding regions (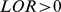, 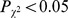). The determination of MREs created by SNPs was performed analogous (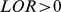, 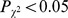). We retrieved 258 miRNAs the targeting of which could be disturbed and 324 miRNAs the formation of mRNA:miRNA hybrids of which could be enhanced.

### Functional Annotation of Genes Containing 3′-UTR SNPs

We evaluated the enrichment of functional annotations of the 326 genes containing 3′-UTR SNPs using DAVID [34], [35] and GSEA [81], [82]. Additionally, the genes were annotated according to their associated traits investigated in the corresponding GWAS. To this end, all traits were mapped to official disease terms as contained in the MeSH (Medical Subject Headings) ontology. As disease class we used the upmost level in the hierarchy tree. Trait enrichment analysis limited to the associated traits as contained in the GWAS Catalog was performed using a 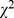 test statistic [80]. For multiple testing correction in Gene Ontology [83] term and disease class enrichment analysis we employed the Bonferroni correction with an overall significance level of 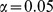.

### Construction of Qualitative Systems Biological Models

For retrieval of phenotype-specific microRNA expression alterations we used PhenomiR [2] and miR2Disease [1]. The interaction data for constructing the network were obtained by manual text-mining and using the public databases IntAct [84], CORUM [85] and KEGG [86]–[88]. Expression data was taken from the MuTHER study [48], made accessible through the Java-interface Genevar [89]. As in the MuTHER study the association of expression values to an allele is given for two separated twin cohorts, we used a combined p-value from both sets to calculate significance. We used Fisher’s combined p-value which was shown to be applicable to expression data [90]. The significance level was adjusted using the rough false discovery rate [91].

## Supporting Information

Table S1 **SNPs predicted to affect 3′-UTR splicing.** The first column lists the SNP rs-numbers, in the second column the respective transcripts are given and the third column contains the genomic locus of the SNP including strand information of the transcripts. The fourth column shows the conservation of the SNP in binary code, i.e. 1 means conserved, 0 means not conserved. The fifth column gives the type of the gained splice site, the score column contains the likelihood of NNSplice and the last column provides the percentage of lost RBP binding sites for the accordant transcripts.
(XLS)

Table S2 **SNPs predicted to affect 3′-UTR secondary structure.** The first column lists the SNP rs-numbers, in the second column the respective transcripts are given and the third column contains the genomic locus of the SNP including strand information of the transcripts. The fourth column shows the conservation of the SNP in binary code, i.e. 1 means conserved, 0 means not conserved. The fifth column lists the correlation coefficient of the wild-type structure to the mutated structure of the respective transcripts.
(XLS)

Table S3 **SNPs disrupting existing MREs.** The first column lists the SNP rs-numbers, in the second column the respective transcripts are given and the third column contains the genomic locus of the SNP including strand information of the transcripts. The fourth column shows the conservation of the SNP in binary code, i.e. 1 means conserved, 0 means not conserved. The fifth column lists the miRNAs the MREs of which are affected.
(XLS)

Table S4 **SNPs creating new MREs.** The first column lists the SNP rs-numbers, in the second column the respective transcripts are given and the third column contains the genomic locus of the SNP including strand information of the transcripts. The fourth column shows the conservation of the SNP in binary code, i.e. 1 means conserved, 0 means not conserved. The fifth column lists the miRNAs for which MREs are created.
(XLS)
